# The Effect of Practice toward Do-Not-Resuscitate among Taiwanese Nursing Staff Using Path Modeling

**DOI:** 10.3390/ijerph17176350

**Published:** 2020-08-31

**Authors:** Li-Fen Wu, Li-Fang Chang, Yu-Chun Hung, Chin Lin, Shiow-Jyu Tzou, Lin-Ju Chou, Hsueh-Hsing Pan

**Affiliations:** 1 Graduate Institute of Medical Sciences, National Defense Medical Center, Taipei City 11490, Taiwan; wulifen@mail.ndmctsgh.edu.tw (L.-F.W.); fang_niki@mail.ndmctsgh.edu.tw (L.-F.C.); 2 Department of Nursing, Tri-Service General Hospital, Taipei City 11490, Taiwan; w51@mail.ndmctsgh.edu.tw; 3 Graduate Institute of Life Sciences, National Defense Medical Center, Taipei City 11490, Taiwan; xup6fup@mail.ndmctsgh.edu.tw; 4 Department of Nursing, Kaohsiung Armed Forces General Hospital, Kaohsiung 80284, Taiwan; A0808020002@mail.802.org.tw; 5 Institute of Medical Science and Technology, National Sun Yat-Sen University, Kaohsiung 80284, Taiwan; 6 School of Nursing, National Defense Medical Center, Taipei City 11490, Taiwan

**Keywords:** do-not-resuscitate, knowledge, attitude, practice, path modeling

## Abstract

This study aimed to elucidate the predictors and the effects of path modeling on the knowledge, attitude, and practice toward do-not-resuscitate (DNR) among the Taiwanese nursing staff. This study was a cross-sectional, descriptive design using stratified cluster sampling. We collected data on demographics, knowledge, attitude, and practice as measured by the DNR inventory (KAP-DNR), Mindful Attention Awareness Scale, General Self-Efficacy Scale, and Dispositional Resilience Scale. Participants were 194 nursing staff from a medical center in northern Taiwan in 2019. The results showed that participation in DNR signature and education related to palliative care were significant positive predictors of knowledge toward DNR. The DNR predictors toward attitude included DNR knowledge, mindfulness, self-efficacy, dispositional resilience, and religious belief of nurses. Generally, the critical predictors of DNR practice were DNR attitude, dispositional resilience, and male nurses. In path modeling, we identified that self-efficacy, dispositional resilience, master’s degree, and religious belief directly influenced practice constituting DNR. Based on the findings of this study, we propose that nurses should improve their self-efficacy and dispositional resilience through training programs. Encouraging staff to undertake further education and have religious beliefs can enhance the practice of DNR and provide better end-of-life care.

## 1. Introduction

The Hospice Palliative Care Act in Taiwan is expressly stipulated to respect terminally ill patients’ will on the medical treatment, and protect their rights. Patients who suffer from severe injury or illness, are diagnosed with an incurable disease, with a fatal prognosis within the near-death and they are allowed to write a letter of intent for the choice of hospice palliative care or life-sustaining treatment [1]. Do-not-resuscitate (DNR) is signed to prevent patients from receiving invalid treatment at the end-of-life or near-death. Such treatments include endotracheal intubation, chest compression, and injection of resuscitation drugs, external defibrillation, artificial cardiac pacing, mouth-to-mouth ventilation, and ventilator use. 

Nursing staffs are the first line of care for patients and play a predominant role in the medical decision-making process, such as the signing of DNR and receiving hospice care for terminal patients and their families. A DNR decision can be a complicated process involving nurses and physicians with a wide variety of experiences and perspectives. A previous study reported that providing DNR information to the patient was important, but a few nurses and physicians stated that they often discuss DNR with patients before issuance [2]. The better the nursing staff’s knowledge and attitude toward the terminal patients’ care, the more confident they can be in discussing DNR and hospice care with terminal patients and their families [3]. DNR discussion should be carried out as early as possible when the patient is relatively healthy and is still able to understand its undertakings, as well as provide a reference for the terminal patient and family members to make medical decisions to provide better care [2,4,5,6].

Additionally, mindfulness emphasizes the attitude of not being critical and allowing its existence to all positive or negative thoughts or emotions [7]. Thus, it can be integrated into the process of DNR discussion, which helps to effectively improve communication and build a good relationship with patients and healthcare professionals [8,9]. Prior studies have identified that mindfulness is correlated with self-efficacy [10,11] and resilience [12]. Self-efficacy is a person’s judgment of their ability to perform a task effectively [13,14]. Resilience is a personality trait that differentiates individuals under stress based on commitment toward life, control of life, and willingness to persist in overcoming challenging obstacles [13]. These factors may affect the nursing staff on facing all kinds of situations and challenges in the line of duty. However, few studies have explored whether these factors are related to the knowledge, attitude, and practice of nursing staff.

Most previous studies have addressed the knowledge, attitude, or practice of DNR or end-of-life decision in physicians [14,15,16,17], or other healthcare providers [18]. Some studies established that physicians and nurses have different end-of-life preferences [19], and DNR decision perspectives for terminal patients [2,20]. However, these prior studies did not explore the knowledge, attitude, and practice of nurses toward the signing of DNR for terminal patients. Multiple regression models are crucial in applying information about the factors that predict knowledge, attitude, and practice (KAP) of nurses toward the signing of DNR. Path analysis holds the strength to study direct and indirect effects concurrently with multiple independent and dependent variables. It is also a method for examining causal patterns among a set of variables [21]. Therefore, the objective of this study was not only to explore the predictors of KAP of nurses toward the signing of DNR but also examine the causal relationship between KAP regarding DNR by using path modeling amongst Taiwanese nursing staff.

## 2. Materials and Methods

### 2.1. Study Design and Population

This study was a cross-sectional design employed in an approximately 1700-bed and 1700-nurse medical center in northern Taiwan from June to August 2019. The medical center was stratified into medical wards, surgical wards, and intensive care units (ICUs). We used a stratified cluster sampling method to recruit nurses. A sample size of 171 was required to undertake *t*-tests and one sample case analysis (using G * power 3.1). We set a two-tailed *p*-value at 0.05, power at 0.90, and effect size at 0.25. We considered an 80% response and completion rate of the questionnaire; therefore, we recruited 207 participants who were registered nurses, able to communicate in Mandarin, and agreed to participate in this study. A total of 13 participants refused to complete the questionnaire. Finally, 194 participants completed the questionnaire.

### 2.2. Instruments

#### 2.2.1. Demographics and Work-Related Characteristics

Demographics collected included age, gender, education level, religious belief, marital status, and the number of children. Work-related characteristic obtained included ward, length of service in nursing, the experience of caring for terminal friends or relatives, participation in DNR signature, frequency of caring for terminal patients (rated on a 5-point scale with 1 being “never” and 5 being “always”), participation in education related to palliative care, participation in training related to DNR.

#### 2.2.2. Knowledge, Attitude, and Practice Regarding DNR Inventory (KAP-DNR)

The KAP-DNR was developed based on the literature review and Hospice Palliative Care Act [1]. The KAP-DNR is composed of 3 scales measuring the knowledge (K-DNR), attitude (A-DNR), and practice (P-DNR) regarding DNR of nurses. 

The K-DNR was used to measure the level of understanding regarding the concept, purpose, and significance of DNR. It contains 15 items; we rated each item as true, false, or I do not know responses. The percentage of correct answer rate was calculated by the number of correct questions divided by the total number of questions.

The A-DNR is composed of 10 items measuring attitudes toward DNR of terminally ill patients. Items 9 and 10 were negatively worded. All items are rated on a 5-point Likert-scale, that is, a 1–5 score with 1 being strongly disagree and 5 strongly agree. The total score ranges from 10 to 50, with a higher score indicating a more favorable attitude toward DNR.

The P-DNR is composed of 10 items measuring the nurse’s ability to communicate with a patient or family caregiver regarding DNR actively. Item 3 was negatively worded, wherein on a 1–5 score, 1 indicated; never, as 5 indicated; always. The total score ranges from 10 to 50, with a higher score indicating more favorable practices regarding DNR. 

Content validity was established by a panel of 5 experts, which consisted of an associate professor, a physician, a supervisor, a head nurse, and a hospice shared care nurse, all working within the field of terminal care or palliative care. Experts rated each item of KAP-DNR on relevance, accuracy, and applicability on a 1–5 score. We counted the content validity index (CVI) for each item depending on the number of experts rating the item on a 4 or 5 score and divided that number by the total number of experts. The average CVI across the items in this study was 0.92. Cronbach’s alpha of the K-DNR, A-DNR, and P-DNR was 0.505, 0.775, and 0.871 for the 194 nurses, respectively.

#### 2.2.3. Mindful Attention Awareness Scale (MAAS)

MAAS was developed by Brown and Ryan (2003) [22] and translated into the Chinese version by Chang, Lin, and Huang (2011) [23]. MAAS measures two unique factors, attention and awareness. This scale contains 15 items, rated on a 1–6 score with 1 being almost not and 6 being always. Items were all negatively worded. The total score ranges from 15 to 90, with a higher score indicating a higher mindfulness level. Internal consistency, Cronbach’s *α*, was 0.81. Cronbach’s α of the MAAS was 0.902 for the 194 nurses in this study.

#### 2.2.4. General Self-Efficacy Scale (GSES)

The GSES was initially developed in German by Jerusalem and Schwarzer in 1981, first as a 20-item version and later as a shorten 10-item version [24]. This scale measures a broad and stable sense of personal competence to deal efficiently with a variety of stressful situations and has been translated into different languages. Jerusalem and Schwarzer developed the Chinese version of the GSES and tested it in a sample of 74 Chinese adults with mild mental health symptoms. This scale was unidimensional with excellent internal reliability, Cronbach’s α = 0.92 [25]. The scale contains 10 items rated on a 1–4 score, with 1 being wholly incorrect and 4 as entirely correct. The total score ranges from 10 to 40, with a higher score indicating a higher self-efficacy to deal with stressful situations. Internal consistency, Cronbach’s α, of the GSES was 0.926 for the 194 nurses in this study.

#### 2.2.5. Dispositional Resilience Scale (DRS)

DRS was developed by Tu and Weng (2013) based on the 15-item Dispositional Resilience Scale [26]. We employed this scale to measure psychological hardiness, considered as a personality style to differentiate individuals under stress based on commitment toward life, control of life, and willingness to overcome challenges. The scale contained 15 items; divided into three subscales, that is, commitment, control, and challenge, wherein, each subscale contained 5 items. The rating each item was on a 0–3 score with 0 being “I do not agree” and 3 as “totally agree”. The total score ranges from 0 to 45, with a higher rating indicating higher hardiness. This scale displayed acceptable reliability and validity [26]. In this study, internal consistency, Cronbach’s *α*, of the overall DRS was 0.871, and the subscales of commitment, control, and challenge were 0.730, 0.811, and 0.701, respectively, for the 194 nurses.

### 2.3. Study Process

We used stratified cluster sampling by medical ward, surgical ward, and ICUs to recruit nurses who fulfilled the inclusion criteria. In the case that a particular ward or unit was selected, the researcher contacted the leader of the ward or unit first. We described the aims and methods of this study for participants in a meeting room at ward meetings. Questionnaires were unidentified and the information was deemed confidential. Participants spent around 15–20 min to fill out the surveys. However, they were permitted to discontinue the study at their discretion. After completing the questionnaire, participants received a gift card. A total of 194 subjects completed the survey. The Institutional Review Board (IRB 2-108-05-066) approved this study.

### 2.4. Statistical Analysis

The collected data was coded using excel and analyzed by IBM SPSS Statistics for Windows, Version 22.0 (IBM Corp., Armonk, NY, USA). In which we described the categorical variables by frequency and percentage. As well, defining the continuous variables by mean and standardized deviation (SD). Multiple linear regression was used to examine the predictors for KAP of nurses toward the signing of DNR. Further, we used path analysis to describe the direct or indirect dependencies among a set of variables, including mindfulness, self-efficacy, resilience, demographics, and work-related characteristics. Our previous study shows the details [27]. Wherein, a *p*-value of <0.05 was considered statistically significant.

## 3. Results

### 3.1. Demographics and Work-Related Characteristics

The mean age of the participants was 29.71 years. Most of the participants were females (92.8%), with bachelor’s degrees (85.1%), no religious beliefs (53.1%), and single (83.0%). The mean length of service in nursing was 6.59 years. The most substantial proportion of the participants working in the ICU was 38.7%, no experience of caring for terminal friends or relatives (68.6%), no participation in DNR signature (72.7%), participated in education related to palliative care (88.1%) and DNR (77.3%). The mean frequency of caring for terminal patients was 3.47 (57.0%) (Table 1).

### 3.2. Knowledge, Attitude, and Practice toward DNR Signature, and Mindfulness, Self-Efficacy, and Dispositional Resilience among Nurses 

The mean percentage of correct answer rate of knowledge toward the DNR signature of the terminal patient among nurses was 73.99 (SD = 9.9). The mean scores of attitude and practice toward the DNR signature of the terminal patient among nurses were 42.53 (SD = 4.46), and 38.30 (SD = 6.25), respectively. The mean scores of mindfulness, self-efficacy, and dispositional resilience were 66.84 (SD = 9.87), 25.25 (SD = 5.14), and 27.49 (SD = 5.10), respectively. Among dispositional resilience scores, nurses scored the highest in control and lowest in the challenges (Table 2).

### 3.3. Predictors of Knowledge, Attitude, Practice toward DNR Signature among Nurses

As shown in Table 3, the significant predictors of the K-DNR among nurses were participation in DNR signature, and participation in education related to palliative care after adjustment for mindfulness, self-efficacy, dispositional resilience, demographics and work-related characteristics among nurses. Nurses who had participated in the DNR signature had a higher mean score for the K-DNR than those who had never participated in DNR signature by 3.88 points (95% CI = 0.26~7.50, *p* = 0.037). Nurses who had participated in education related to palliative care had a higher mean score for the K-DNR than those who had never participated in relevant training by 9.18 points (95% CI = 3.73~14.64, *p* = 0.001).

DNR knowledge, mindfulness, self-efficacy, dispositional resilience, religious belief were important predictors for the A-DNR, especially after adjustment for DNR knowledge, mindfulness, self-efficacy, dispositional resilience, demographics, and work-related characteristics among nurses. Nurses with higher DNR knowledge (β = 0.10, 95% CI = 0.03~0.16, *p* = 0.004), mindfulness (β = 0.08, 95% CI = 0.01~0.14, *p* = 0.019), self-efficacy (β = 0.20, 95% CI = 0.05~0.35, *p* = 0.010), or lower dispositional resilience (β = −0.16, 95% CI= −0.32~−0.01, *p* = 0.039) had better attitude toward DNR. Nurses who have religious beliefs had a higher mean score for the A-DNR than those who had no religious belief by 2.23 points (95% CI = 0.99~3.48, *p* = 0.001).

DNR attitude, dispositional resilience, and male gender were important predictors for the P-DNR. Most so after adjustment for DNR knowledge, DNR attitude, mindfulness, self-efficacy, dispositional resilience, demographics, and work-related characteristics among nurses. Nurses with higher DNR attitude (β = 0.62, 95% CI = 0.42~0.81, *p* < 0.001), or dispositional resilience (β = 0.24, 95% CI = 0.04~0.44, *p* = 0.023) had better practice toward DNR. Male nurses had a higher mean score for the P-DNR than female nurses by 4.10 points (95% CI = 1.01~7.19, *p* = 0.010).

### 3.4. Path Modeling of Knowledge, Attitude, and Practice toward DNR Signature among Nurses

The path modeling demonstrated that self-efficacy, dispositional resilience, master/junior college, and religious beliefs directly affected practice. The relationships between self-efficacy (Coefficients = 0.272, *p* < 0.001), dispositional resilience (Coefficients = 0.202, *p* = 0.006), master/junior college (Coefficients = 0.149, *p* = 0.040), and religious belief (Coefficients = 0.155, *p* = 0.033) were significant by standardized coefficient estimates for the paths. The above data are shown in Table 4.

## 4. Discussion

### 4.1. Factors Affecting the KAP-DNR among Nurses

This study found that participation in DNR signature and participation in education related to palliative care were positive predictors of knowledge toward the DNR signature of the terminal patient among nurses. Most prior studies indicate similar results. A study in Taiwan showed that a higher level of understanding toward palliative care consultation service among nurses was associated with participation in education related to palliative care [27]. Having a bachelor of science or a higher degree in nursing, working in an emergency department, having a daily experience of caring for chronically ill patients, and taking part in a training on the end-of-life care were significantly associated with good knowledge of nurses toward the end-of-life care [28]. Nurses with a poor understanding of palliative care were the main limitation to providing excellent palliative care [3]. The level of knowledge of nurses as well as experience in advance directives (ADs) discussions reported a moderate level of confidence to explain ADs to patients and their families [29]. Nurses can improve their knowledge through in-service education and on job retraining to promote the quality of palliative care services for the patients [3]. Therefore, it is crucial to participate in the DNR signature with patients or family and receive education related to palliative care to improve the nurses’ knowledge toward DNR.

This study established that the DNR knowledge, mindfulness, self-efficacy, dispositional resilience, and religious beliefs were significant positive predictors of the attitude of nurses toward the DNR signature. A study reported that the knowledge level of nurses has an impact on their confidence to discuss end-of-life care decisions with patients and their families [29]. A mixed-method systematic review indicated that mindfulness could improve nurses’ mental health and promote performance at work, such as better communication with patients, higher sensitivity to patients’ conditions, precise analysis of complex situations, and emotional regulation in stressful situations [9]. The self-efficacy of nurses showed a significantly positive correlation with end-of-life care attitude. Self-efficacy is essential in fostering confidence in nurses to help patients and their families make appropriate medical decisions and to provide proper end-of-life care [30]. Dispositional resilience is as hardiness, a protective factor against perceived stress, and a facilitating factor for happiness in nurses [31]. Nurses with resilience were more likely to report better quality of care and a higher personhood status [32]. A study in Palestinians asserted that professionals’ attitudes toward DNR were highly influenced by their religious and cultural background [20]. Religion plays a vital role in the lives of many people, such that spiritual and ethical issues are usually aroused or strengthened as patients near the end-of-life [33]. Thus, these factors, DNR knowledge, mindfulness, self-efficacy, dispositional resilience, and religious beliefs might influence the attitude of nurses toward the DNR signature. We suggest that nurse training programs such as simulation workshop might be an effective strategy to improve nursing knowledge, mindfulness, self-efficacy, and resilience.

This study showed that DNR attitude, dispositional resilience, and male nurses were significant positive predictors of the practice of nurses toward DNR. These findings were similar to prior studies. Nurses with precise knowledge and positive attitude toward end-of-life care are essential in raising confidence in nurses to communicate actively with patients and their families and help them make suitable medical decisions and to provide appropriate end-of-life care [30,34]. Dispositional resilience refers to the psychological resilience that can enable nurses to effectively face stress when signing DNR for families of end-stage patients [35]. This situation is exclusive in male nurses because they have a higher confidence level to actively discuss DNR with patients or family caregivers [36]. Therefore, we suggest that a brief teaching intervention can help nurses take patient preferences at the end-of-life care [37]. In addition, there are some strategies that can cultivate nurse resilience such as facilitating social connections, encouraging nurses’ self-care, and fostering mindfulness practice [38]. 

### 4.2. Path Modeling of Knowledge, Attitude, and Practice

The results of our path analysis indicated that self-efficacy, dispositional resilience, master/junior college, and religious belief directly affected the practice of DNR signature among nurses. 

Many previous studies have demonstrated that self-efficacy is correlated with practice [39,40]. The main reason is that self-efficacy can improve nurses’ professional practice behaviors and make them feel confident to deliver the appropriate, timely, and compassionate care [40,41]. Dispositional resilience had a positive correlation with hardiness, self-esteem, life, and job satisfaction. It was also a protective factor against perceived stress and a facilitating factor for happiness in nurses [31,42]. The higher the dispositional resilience level, the lower the burnout situations in nurses who can successfully overcome obstacles, uncertainties, and adverse conditions [43]. Simulation workshops have been indicated as an effective strategy to improve self-efficacy and resilience [44]. We suggest that nurses can provide a better quality of care in nursing practice by these training programs.

Having a master’s degree and religious belief also had a positive direct effect on nurses’ practice toward the DNR signature of family caregivers of the terminal patients. This finding was similar to a prior study that holders of a master’s degree indirectly and positively influenced good practice toward palliative care [27]. Higher educational levels among nurses meant having more confidence to deal with patients’ problems in clinical nursing practices [45]. Prior studies have shown that religious beliefs are a significant factor in forming caregivers’ and health care providers’ viewpoints about DNR decisions [46,47]. This significance is because religious beliefs play an essential role in the lives of many people since spiritual and ethical issues are usually aroused or strengthened as patients near end-of-life, as well as nurses on nursing practice behaviors toward the end-of-life care [33]. Therefore, we encourage nurses to undertake further education and have religious beliefs to enhance the practice of DNR and provide better end-of-life care. 

### 4.3. Limitations and Recommendations

This study has several limitations. First, our study population was limited to one medical center, the generalizability of our findings is limited. Second, the KAP-DNR was a nurse-reported assessment tool. This might be over-reported by nurses and the exact DNR signature rate of caregivers of the terminal patients was unknown. Finally, this study used a cross-sectional design, the change in KAP-DNR overtime in nursing staff was not explored. 

Based on the limitations of this study, future research suggestions are indicated as follows: First, samples from different centers in Taiwan are needed to confirm our findings. Second, the use of extra empirical assessment tools and the calculation of the signature rate of DNR are needed to determine the DNR practice level in nurses. Finally, studies using a longitudinal study design are needed to explore the trend in KAP-DNR among nurses.

## 5. Conclusions

This study suggests that participation in DNR signature and participation in education related to palliative care were significant predictors of knowledge toward the DNR. DNR knowledge, mindfulness, self-efficacy, dispositional resilience, and religious beliefs were significant predictors of attitude toward DNR. Finally, DNR attitude, dispositional resilience, and male nurses were the critical predictors of practices toward the DNR. Additionally, self-efficacy, dispositional resilience, master’s degree, and religious beliefs directly influenced the practice regarding DNR. Based on the findings of this study, we suggest that nurses should improve their self-efficacy and dispositional resilience by training programs as well as receiving advanced education in nursing and having religious beliefs. Fulfilling the suggestions may allow nurses to appreciate the importance of practice toward DNR signature, and enhance the quality of care for the terminal patients receiving end-of-life care.

## Figures and Tables

**Table 1:** Demographics and work-related characteristics (*N* = 194).

Variables	*N* (%)/Mean ± SD
Demographics	
Age(year)	29.71 ± 6.70
Gender	
Female	180 (92.8%)
Male	14 (7.2%)
Education level	
Junior college	23 (11.9%)
Bachelor	165 (85.1%)
Master	6 (3.1%)
Religious Belief	
No	103 (53.1%)
Yes	91 (46.9%)
Marital Status	
Single	161 (83.0%)
Married	33 (17.0%)
**Work-related characteristics**	
Ward	
HemaOnco	21 (10.8%)
Medical	29 (14.9%)
Surgical	48 (24.7%)
ICU	96 (49.5%)
Length of service in nursing (year)	6.59 ± 6.00
Experience of caring for terminal friends or relatives	
No	133 (68.6%)
Yes	61 (31.4%)
Participation in DNR signature	
No	141 (72.7%)
Yes	53 (27.3%)
Frequency of caring for terminal patients	3.47 ± 0.78
Participation in education related to palliative care	
No	23 (11.9%)
Yes	171 (88.1%)
Participation in training related to DNR	
No	44 (22.7%)
Yes	150 (77.3%)

SD Standard deviation; DNR Do-not-resuscitate; HemaOnco Hematology and oncology; ICU Intensive care unit.

**Table 2:** Knowledge, attitude, and practice toward do-not-resuscitate (DNR) signature, and mindfulness, self-efficacy, and dispositional resilience among nurses (*N* = 194).

Variables	Mean ± SD
Knowledge	11.10 ± 1.48
Percentage of correct answer	73.99 ± 9.90
Attitude	42.53 ± 4.46
Practice	38.30 ± 6.25
Mindfulness	66.84 ± 9.87
Self-efficacy	25.25 ± 5.14
Dispositional resilience	27.49 ± 5.10
Commitment	9.23 ± 1.98
Control	9.59 ± 2.06
Challenge	8.67 ± 2.01

DNR Do-not-resuscitation; SD Standard deviation.

**Table 3:** Predictors of knowledge, attitude, and practice toward DNR signature among nurses (*N* = 194).

	Knowledge		Attitude		Practice	
Variables	Adjusted β (95% CI)	*p* Value	Adjusted β (95% CI)	*p* Value	Adjusted β (95% CI)	*p* Value
**Knowledge**	--	--	0.10 (0.03–0.16)	0.004	−0.02 (−0.11–0.07)	0.671
**Attitude**	--	--	--	--	0.62 (0.42–0.81)	<0.001
**Practice**	--	--	--	--	--	--
**Mindfulness**	0.19 (0.04 - 0.33)	0.014	0.08 (0.01–0.14)	0.019	−0.06 (−0.14–0.03)	0.204
**Dispositional resilience**	0.10 (−0.25−0.46)	0.568	−0.16 (−0.32–−0.01)	0.039	0.24 (0.04–0.44)	0.023
**Self-efficacy**	0.09 (−0.26–0.43)	0.627	0.20 (0.05–0.35)	0.010	0.07 (−0.13–0.26)	0.501
**Demographics**						
Age (years)	−0.06 (−0.59–0.48)	0.839	−0.13 (−0.37–0.10)	0.275	−0.10 (−0.40–0.20)	0.519
Gender						
Female	Reference		Reference		Reference	
Male	3.68 (−1.75–9.11)	0.186	−1.91 (−4.28–0.47)	0.118	4.10 (1.01–7.19)	0.010
Education level						
Junior college	Reference		Reference		Reference	
Bachelor	3.33 (−1.11–7.77)	0.143	0.21 (−1.73–2.16)	0.830	−0.58 (−3.09–1.93)	0.650
Master	7.74 (−1.73–17.21)	0.111	2.68 (−1.47–6.84)	0.207	3.04 (−2.35–8.42)	0.271
Religious belief						
No	Reference		Reference		Reference	
Yes	0.86 (−2.00–3.73)	0.556	2.23 (0.98–3.48)	0.001	0.38 (−1.29–2.05)	0.654
Marital status						
Single	Reference		Reference		Reference	
Married	−0.18 (−5.23–4.87)	0.945	0.24 (−1.96–2.44)	0.830	0.89 (−1.95–3.73)	0.541
**Work-related characteristics**						
Ward						
HemaOnco	Reference		Reference		Reference	
Medical	−0.19 (−5.79–5.42)	0.948	0.27 (−2.17–2.71)	0.829	0.50 (−2.65–3.65)	0.756
Surgical	−1.09 (−6.32–4.15)	0.685	1.54 (−0.74–3.82)	0.187	0.60 (−2.36–3.55)	0.692
ICU	1.40 (−3.43–6.22)	0.572	0.23 (−1.87–2.33)	0.832	−0.23 (−2.95–2.48)	0.867
Length of service in nursing	0.06 (−0.57–0.69)	0.852	0.06 (−0.21–0.34)	0.647	0.10 (−0.25–0.46)	0.564
Experience of caring for terminal friends or relatives						
No	Reference		Reference		Reference	
Yes	−0.72 (−4.21–2.77)	0.687	0.76 (−0.76–2.28)	0.327	−0.65 (−2.62–1.31)	0.516
Participation in DNR signature						
No	Reference		Reference		Reference	
Yes	3.88 (0.26–7.50)	0.037	−0.19 (−1.79–1.41)	0.815	0.72 (−1.34–2.78)	0.495
Frequency of caring for terminal patients	0.76 (−1.15–2.67)	0.437	0.73 (−0.10–1.57)	0.087	0.67 (−0.42–1.76)	0.228
Participation in education related to palliative care						
No	Reference		Reference		Reference	
Yes	9.18 (3.73–14.64)	0.001	1.61 (−0.84–4.06)	0.201	−0.38 (−3.56–2.80)	0.814
Participation in training related to DNR						
No	Reference		Reference		Reference	
Yes	−2.60 (−6.63–1.44)	0.209	−0.81 (−2.58–0.95)	0.369	0.91 (−1.38–3.19)	0.437

HemaOnco Hematology and oncology; ICU Intensive care unit; CI Confidence interval; DNR Do-not-resuscitate.

**Table 4:** Path modeling for knowledge, attitude, and practice toward DNR among nurses (*N* = 194).

	Knowledge→Attitude→Practice		Attitude→Practice		Practice	
Variables	Coefficients	*p* Value	Coefficients	*p* Value	Coefficients	*p* Value
**Mindfulness**	<0.001	1.000	<0.001	0.995	0.070	0.333
**Self-efficacy**	−0.001	0.987	0.088	0.225	0.272	<0.001
**Dispositional Resilience Scale**	−0.001	0.986	0.094	0.192	0.202	0.006
**Demographics**						
Age	<0.001	1.000	0.003	0.969	−0.005	0.945
Gender						
Female/Male	−0.001	0.990	0.070	0.330	0.130	0.073
Education level						
Bachelor/Junior college	−0.001	0.994	−0.042	0.559	−0.096	0.185
Master/Junior college	<0.001	0.997	0.047	0.512	0.149	0.040
Religious Belief						
Yes/No	<0.001	0.997	0.023	0.751	0.155	0.033
Marital Status						
Married/Single	<0.001	0.996	0.047	0.518	0.112	0.122
**Work-related characteristics**						
Ward						
Medical/HemaOnco	<0.001	0.998	0.020	0.784	0.035	0.628
Surgical/HemaOnco	<0.001	1.000	−0.005	0.948	0.007	0.923
ICU/HemaOnco	<0.001	1.000	0.015	0.835	0.029	0.688
Length of service in nursing	<0.001	0.999	0.008	0.907	0.005	0.945
Experience of caring for terminal friends or relatives						
Yes/No	<0.001	0.999	0.007	0.922	0.054	0.455
Participation in DNR signature						
Yes/No	<0.001	1.000	<0.001	0.995	0.011	0.879
Frequency of caring for terminal patients	<0.001	0.999	0.014	0.842	0.105	0.147
Participation in education related to palliative care						
Yes/No	<0.001	0.999	0.026	0.722	0.138	0.057
Participation in training related to DNR						
Yes/No	<0.001	0.999	0.036	0.620	0.100	0.167

HemaOnco Hematology and oncology; ICU Intensive care unit; DNR Do-not-resuscitate.
